# Association of Influenza Virus Proteins with Membrane Rafts

**DOI:** 10.1155/2011/370606

**Published:** 2011-07-25

**Authors:** Michael Veit, Bastian Thaa

**Affiliations:** Department of Immunology and Molecular Biology, Veterinary Faculty, Free University Berlin, Philippstraße 13, 10115 Berlin, Germany

## Abstract

Assembly and budding of influenza virus proceeds in the viral budozone, a domain in the plasma membrane with characteristics of cholesterol/sphingolipid-rich membrane rafts. The viral transmembrane glycoproteins hemagglutinin (HA) and neuraminidase (NA) are intrinsically targeted to these domains, while M2 is seemingly targeted to the edge of the budozone. Virus assembly is orchestrated by the matrix protein M1, binding to all viral components and the membrane. Budding progresses by protein- and lipid-mediated membrane bending and particle scission probably mediated by M2. Here, we summarize the experimental evidence for this model with emphasis on the raft-targeting features of HA, NA, and M2 and review the functional importance of raft domains for viral protein transport, assembly and budding, environmental stability, and membrane fusion.

## 1. Introduction

### 1.1. Influenza Viruses: Molecular Composition

**Figure 1:**
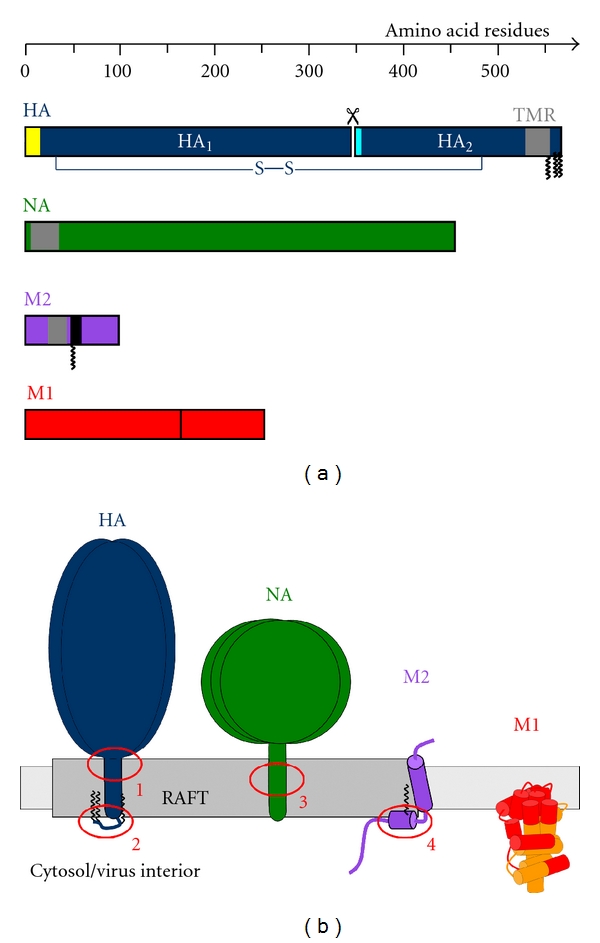
The membrane-associated proteins of influenza virus and their raft association. (a) Primary amino acid sequence of hemagglutinin (HA, processed into HA_1_ and HA_2_, blue), neuraminidase (NA, green), M2 (purple), and M1 (red, with amphiphilic helix in black). Transmembrane regions (TMR) in gray, S-acylations in HA and M2 indicated by zigzag line, signal peptide of HA in white, and fusion peptide in HA in cyan. (b) Topology of HA, NA, M2, and M1 in the membrane, raft localization indicated. Raft-targeting features: (1) hydrophobic amino acids in the outer part of the HA-TMR; (2) S-acylation of HA; (3) outer part of the NA-TMR; (4) S-acylation and cholesterol binding of the amphiphilic helix of M2. M1 according to the structure model of Shishkov et al. [1], membrane-interactive regions in red. Only one monomer of the trimeric HA and the tetrameric NA and M2 is shown for clarity.

Influenza virus particles are heterogeneous in shape, either spherical (with a diameter of roughly 100 nm) or filamentous (with a length of several micrometers). The particles contain the viral genome, which is segmented into eight entities termed viral ribonucleoprotein particles (vRNPs), each composed of a segment of viral RNA complexed to the nucleoprotein (NP) and the subunits of the viral RNA polymerase (PA, PB1, and PB2). The vRNPs are encased by a protein layer consisting of the matrix protein M1, which also lines the viral envelope from beneath and is supposed to bind to all other viral constituents. The viral envelope is a lipid bilayer derived from the apical plasma membrane of the infected cell. There are three transmembraneous viral proteins embedded in the envelope: the glycoproteins hemagglutinin (HA) and neuraminidase (NA), which protrude at the viral surface as “spikes,” and—in minor quantities—the proton channel protein M2. Here, we will focus on the buildup of the viral envelope and the proteins involved (HA, NA, M2, M1), which are depicted in Figure 1.

**HA** (blue in Figure 1) is a type I transmembrane protein with an N-terminal signal peptide (white in Figure 1(a)), which is cleaved off after cotranslational sequestration of the nascent polypeptide chain into the endoplasmic reticulum (ER), a large ectodomain (positioned in the ER lumen and towards the extracellular milieu when located at the plasma membrane), a single transmembrane region (TMR) of approximately 27 amino acid residues located near the C-terminus of the protein, and a short cytoplasmic tail (approximately 11 residues). 

HA assembles into a homotrimer in the ER and is transported *via* the secretory pathway to the plasma membrane, more specifically the apical plasma membrane in polarized (e.g., epithelial) cells, where virus assembly and budding take place [2]. In the ER and Golgi, HA is glycosylated in the ectodomain, and typically three saturated fatty acid chains are covalently attached to C-terminal cysteine residues (S-acylation). The first cysteine residue, at the border between TMR and cytoplasmic tail, is modified with stearate, while the other two cysteines in the cytoplasmic tail carry palmitates [3, 4].

The large ectodomain is processed into two subunits (HA_1_ and HA_2_) by a protease provided by the host organism; they remain linked by a disulfide bridge [5]. This proteolytic maturation is needed to enable *membrane fusion*, which is exerted by HA during virus entry: a hydrophobic part termed “fusion peptide” (cyan in Figure 1(a)) becomes exposed at the N-terminus of HA_2_ after cleavage and is inserted into the host endosomal membrane upon activation of HA (conformational change of the ectodomain by low pH). Apart from membrane fusion, HA is also responsible for receptor recognition: a binding pocket in HA_1_ recognizes sialic acid moieties in glycoproteins and glycolipids on the host cell surface [6].

**NA** (green in Figure 1) is a type II transmembrane protein, which assembles into a homotetramer. The first five residues from the N-terminus form the cytoplasmic tail, followed by a transmembrane anchor encompassing approximately 30 residues and the glycosylated ectodomain. NA is processed along the same intracellular route as HA (ER-Golgi-apical plasma membrane). The main function of the protein is cleavage of terminal sialic acid moieties from glycans in the mucus of the host's respiratory tract and on the viral surface, thus helping in release of newly formed viral particles from the host cell. NA is the target of the anti-influenza drug oseltamivir and other neuraminidase inhibitors [7].

**M2**, the third viral transmembrane protein (purple in Figure 1), is also tetrameric and forms a proton channel activated by acidic pH, the action of which is important for genome unpacking during virus entry and can be inhibited—at least in Influenza A virus strains—by the drug amantadine. In each monomer, the first 24 amino acids form the ectodomain, which is not glycosylated, the following 19 residues are the transmembrane region, and the remaining 54 residues build up the cytoplasmic tail. The sequence immediately following the TMR shapes a membrane-parallel amphiphilic helix, an *α*-helix with a hydrophobic face (which partially protrudes into the membrane), and a hydrophilic face (which points to the cytosol). A cysteine residue in this part of the protein is S-acylated. Similarly to HA and NA, M2 is transported along the secretory pathway to the apical plasma membrane, but the apical targeting signal in M2 has not been identified. Contrary to the other viral transmembrane proteins, however, M2 is largely excluded from virus particles [8].

The **matrix protein M1** (red in Figure 1) binds to membranes, but does not have a transmembrane span [9–11]. M1 is most likely attached to membranes by extended regions of the protein or by the cooperative action of several binding sites [12–16]. Concomitantly, a large portion of the protein was found to be membrane-associated by mass-spectrometry-based structural reconstruction [1]. M1 is central for virus morphogenesis as it is supposed to bind to all other viral components including the vRNPs and the membrane. However, M1 is not intrinsically targeted to the assembly site, the apical plasma membrane, but rather localizes to the nucleus, internal membranes, and the cytosol. Plasma membrane targeting of M1 is therefore in all likelihood mediated by at least one of the viral transmembrane proteins (HA, NA, and M2). The interaction of M1 with the cytoplasmic tails of HA (11 residues) and NA (5 residues) has only been demonstrated indirectly, for instance, by altered detergent solubility [17, 18] or increased membrane association [19] of M1 in the presence of HA/NA, an effect not seen in all studies [15]. In contrast, the interaction between M1 and the cytoplasmic tail of M2 (54 residues) is well documented, most conclusively by coimmunoprecipitation [20, 21]. M1 has the capacity to oligomerize [16], which is supposed to cluster the viral components together at the site of virus budding to organize the assembly process.

### 1.2. Biochemical and Biophysical Properties of Membrane Rafts

Contrary to the classical view of the plasma membrane as a homogenous lipid mixture, there is now conclusive evidence indicating the presence of specialized lipid domains with distinct biochemical and biophysical properties. These *membrane rafts* are dynamic assemblies of cholesterol, sphingolipids, and phospholipids containing saturated fatty acids. Sphingolipids are exclusively present in the external leaflet of the plasma membrane, whereas the composition of inner leaflet rafts is not known, but it has been suggested that cholesterol plus phospholipids with saturated acyl chains is enriched [22, 23]. 

Membrane rafts have been characterized extensively in model membrane systems. In the cholesterol/sphingolipid-rich phase, the (mostly saturated) fatty acid chains of the membrane lipids are densely packed and restricted in mobility, but still able to diffuse and rotate, and form a “liquid-ordered” (Lo) phase segregated from the “liquid-disordered” (Ld), more fluid membrane phase. Upon phase separation of Lo and Ld domains, there is a hydrophobic mismatch and a height difference between the two membrane phases, leading to the formation of a “line tension” at their interface. This is conceptionally comparable to surface tension in a three-dimensional system, which—for instance—leads to the formation of a spherical drop of water on an oily surface to minimize the contact area with the repellent surface. Accordingly, line tension leads to the formation of a curved raft phase due to the propensity of the system to minimize its free energy [24].

However, no large-scale, long-lasting phase separation is observed in the membranes of undisturbed cells—yet, highly dynamic (millisecond range) and very small (10–200 nm) heterogeneous membrane organization dependent on the presence of cholesterol has been observed in a plethora of investigations using biophysical methodology of high temporal and spatial resolution [25].

Raft domains are best described for the plasma membrane, although cholesterol/sphingolipid-rich domains are likely to build up already in the Golgi. There are raft-targeting features in proteins for their association with rafts, most decisively glycosyl-phosphatidylinositol (GPI) anchors and S-acylation of cysteine residues [26]. Under certain conditions, for instance, upon ligand binding and receptor oligomerization, the highly dynamic “resting state” rafts can be coalesced and stabilized to fulfill a biological function [25, 27]. One example for functionalization of raft domains are signal transduction processes, for example, the formation of the immunological synapse in the activation of T cells [28]. The assembly and budding of some viruses such as influenza virus is also coupled to the formation of functionalized raft domains, here termed “budozone.” In this context, rafts provide a platform to concentrate the viral components and to facilitate their interactions, while cellular proteins are excluded [24, 25].

## 2. Association of Influenza Virus Proteins with Rafts and Methods to Analyze Them

Influenza virus assembly and budding is linked to (coalesced) membrane rafts in the apical plasma membrane. Generally, the spike glycoproteins HA and NA are assumed to be raft-associated, while M2 is believed to be intrinsically excluded from rafts. The peripheral membrane protein M1 is considered not to have membrane subdomain specificity. In the next paragraphs, we describe the experimental evidence that had led to this model.

### 2.1. Hemagglutinin Is Present in Detergent-Resistant Membranes

Historically, the raft hypothesis was introduced by the observation that some parts of biological membranes (enriched in cholesterol and sphingolipids) resist solubilization by nonionic detergents such as Triton X-100 on ice and float to a low buoyant density upon density gradient centrifugation. These “detergent-resistant membranes” (DRMs) have been considered to be the biochemical correlate for rafts. They have been found to contain proteins, which were hence termed DRM-associated and regarded as raft proteins. In addition, association with DRMs should be sensitive to cholesterol extraction and inhibitors of sphingolipid synthesis [26, 27]. 

The hemagglutinin (HA) of influenza virus was one of the first proteins described as a component of DRMs [29] and has since been judged as paradigm for a raft-associated transmembrane protein. 

The detergent extraction method was used in combination with mutagenesis to identify molecular signals in HA for raft localization. Alanine scanning of the whole transmembrane region (an exchange of three consecutive amino acids at a time by alanines) showed that hydrophobic residues located at the outer leaflet of the membrane bilayer are responsible for resistance against detergent extraction [29, 30], see label 1 in Figure 1(b). In addition, S-acylation at cytoplasmic and transmembrane cysteine residues (label 2) are required for partitioning of HA into DRMs [30]. From detergent-extraction experiments, it was concluded that palmitate bound to the cytoplasmic cysteines is more important for raft association than the stearate attached to the transmembrane cysteine [31, 32]. 

Presently, one can only speculate on the molecular mechanism by which the raft-targeting signals cause incorporation of HA into rafts. In principle, *α*-helical transmembrane regions with their protruding amino acid side chains should rather disrupt the tight packing of lipids in a raft domain as they do not readily accommodate the rigid, bulky sterol ring of cholesterol. However, direct binding of cholesterol to the protein could lead to raft targeting. In motifs such as the “cholesterol recognition/interaction amino acid consensus” (CRAC, [33]) or the “cholesterol consensus motif” (CCM, [31]), a large aliphatic residue (valine or isoleucine), a tyrosine or phenylalanine residue, and a basic amino acid (arginine or lysine) coordinate the cholesterol moiety if positioned accordingly. It is conceivable that the raft-targeting residues in the HA-TMR (valine-isoleucine-leucine/VIL), two of which (IL) are strictly conserved across all HA subtypes, form part of a cholesterol interaction pocket. However, since atomic structural information of the HA-TMR is still lacking, it is unclear whether the amino acids in question are ideally positioned. Binding to cholesterol might target HA to preexisting rafts as predicted in the “lipid shell” model for raft association of proteins [32]. Alternatively, it has recently been shown that a peptide representing the transmembrane region of HA induces highly ordered domains in model membranes, but only if the conserved leucine residue is present [34]. Assuming a similar mode of action of the TMR in cellular membranes, this would imply that HA induces the formation of its own raft domains. Furthermore, the substitution of hydrophobic TMR residues by less hydrophobic alanines might shorten the length of the transmembrane span. A long TMR might be required for partitioning of HA into rafts, which are thicker compared to other membrane regions due to stretching of the lipids' fatty acid tails. 

The presence of the second raft-targeting feature in HA, S-acylation, seems to be a common principle in many raft proteins [35]. It could be imagined that flexible acyl chains, especially if attached to the beginning of the transmembrane region, fill the voids in the irregular and rough surface of the transmembrane domain and thus “lubricate” the region for subsequent interactions with cholesterol. In addition, fatty acids attached to the cytoplasmic tail of HA might attract cholesterol, as suggested in the crystal structure of the *β*-adrenergic receptor, in which cholesterol is visible in the vicinity of covalently bound palmitate. However, it should be noted that S-acylation *per se*, irrespective of whether palmitate or stearate is attached, is not sufficient to cause raft localization of viral transmembrane proteins [36]. An example is the HEF glycoprotein of influenza C virus, which does not associate with DRMs, but is stearoylated at a transmembrane cysteine [3, 12].

It has been questioned repeatedly whether association with DRMs reflects raft association of a protein in living cells. Components might be enriched in DRMs simply because they possess common biophysical properties. Furthermore, partitioning of proteins into detergent-soluble and -insoluble fractions is seldom absolute; sometimes, only very few percent of a protein population are present in DRMs. Extraction conditions are not standardized, and therefore results obtained by using different protocols can hardly be compared [37]. Thus, more sophisticated methods have subsequently been used to confirm and characterize the raft localization of HA.

### 2.2. Analysis of the Distribution of HA with High-Resolution Methods

Fluorescence microscopy in living cells has failed to reveal laterally segregated clusters of HA or other raft-associated proteins and lipids, indicating that rafts in undisturbed cells must be smaller than the resolution of the light microscope (<200 nm). However, when antibodies against both HA and a glycolipid-anchored protein were applied, HA was found to cocluster with the established raft component. It was assumed that cross-linking of HA trimers by antibodies stabilizes small raft structures, which subsequently coalesce with other raft domains to form large, visible patches [38]. However, this study could not establish potential clustering of HA in unperturbed cells. Since then, methods with a higher lateral resolution than the conventional light microscope have been used. 

Immunoelectron microscopy combined with rigorous statistical analysis showed that in transfected cells HA neither is randomly distributed in the plasma membrane nor accumulates only in very small domains with the size of a small, dynamic raft. Instead, clustering of HA molecules on different length scales from 20 nm up to 900 nm was found. In the course of virus infection, the HA clusters were observed to increase in size, indicating increasing coalescence of HA-containing domains. Since the nanodomains contained the ganglioside GM1, they are likely derived from rafts. However, only HA clusters at the nanometer length scale, that is, with the size of uninduced rafts, could be disintegrated by extraction of cholesterol [39, 40]. Fluorescence-photoactivation-localization microscopy (FPALM), a recently developed nanoscopic method with very high spatial resolution (40 nm), essentially confirmed HA clustering for unfixed, living cells [41]. 

Thus, HA somehow accumulates in microdomains of the membrane or even induces their formation, which leads to its separation from most cellular proteins. Since the area of the HA cluster is large enough to cover the surface of a spherical virus particle, these structures were termed the viral preenvelope or the viral budozone to indicate that they might be a precursor of budding virus particles [39, 42]. The large size of the HA clusters suggests that they are composed of several small rafts. In addition, the HA clusters do not possess round (perimeter minimized), but irregular domain boundaries with long and narrow extensions. This suggests that not only partitioning of HA into rafts, but also other mechanisms for formation of membrane subdomains may be responsible for clustering of HA [39, 43]. One candidate for such a function is cortical actin, which generates a meshwork of microfilaments at the inner leaflet of the plasma membrane that might transiently entrap HA [44]. Furthermore, cortical actin might organize and maintain the formation of rafts [45]. This effect might be mediated by the lipid phosphatidylinositol-(4,5)-phosphate (PtdIns(4,5)P_2_), a key regulator of assembly and disassembly of microfilaments [46]. The actin cytoskeleton has also been shown to be functionally linked to rafts in the context of virus infection: the disruption of the actin meshwork leads to a redistribution of HA and to reduced budding, most relevantly in the case of filamentous virus particles, maybe because their surface area is higher than in spherical particles [38].

Förster's (or fluorescence) resonance energy transfer (FRET) is exceptionally well suited to demonstrate very close association between two molecules, for example, if they populate the same small raft domain, even for a very short time period. FRET relies on the transfer of energy from an excited donor fluorophore, such as the cyan-fluorescent protein (CFP), to an acceptor fluorophore, for example, the yellow-fluorescent protein (YFP), if they are in very close vicinity to each other (<10 nm). Association of two proteins can be assessed in cells by fusing them to CFP and YFP, respectively, and conducting FRET measurements. To exclude that a FRET signal is due to random collision of two molecules, as it may occur frequently if both diffuse in the plane of a membrane, the FRET data obtained must be correlated with the expression level of the probes. If the FRET efficiency increases with the concentration of the FRET acceptor protein at the membrane, FRET is caused by random collision. In contrast, if FRET is due to clustering of the two proteins under study, the FRET efficiency is largely independent of the concentration of the acceptor protein and saturated even at relatively low FRET acceptor concentrations [39, 40]. To evaluate the data, a hyperbolic function is fitted to the data, which yields a “dissociation constant” *K_D_* as a parameter to assess the associative properties of donor and acceptor [43]. Influenza virus HA, fused at its cytoplasmic tail to CFP [42], clusters with an established marker for inner leaflet rafts, myristoylated and palmitoylated YFP [43]. Furthermore, an artificial HA-derived FRET probe, consisting of a signal peptide, a fluorescent protein, and the transmembrane as well as cytoplasmic domain of HA [44], clusters with a glycolipid-anchored protein, an established marker for rafts of the outer leaflet. In this construct, tagging of the cytoplasmic tail was circumvented to avoid interference with its role in lateral organization. For both HA constructs, clustering was significantly reduced when rafts were disintegrated by cholesterol extraction and when the two described raft-targeting signals of HA were removed. Both signals had a similar effect on raft-targeting of HA and did not work synergistically with each other. 

One disadvantage of the FRET technique is that neither *K_D_* values nor FRET-efficiencies can be compared between different protein pairs, even if they are attached to the same donor and acceptor fluorophore. The FRET efficiency depends on the distance between the donor and the acceptor and their relative orientation, parameters which cannot be measured within cells. It is thus not possible to determine the percentage of molecules that interact with a raft marker or quantitatively compare the raft association of different viral proteins.

### 2.3. Diffusional Mobility of HA at the Plasma Membrane

It has been hypothesized that the embedding of a protein in raft domains leads to a slower diffusion compared to non-raft proteins, which diffuse as single entities [47]. Accordingly, fluorescence recovery after photobleaching (FRAP), where the speed of replenishment of a previously bleached spot within the membrane is measured, was employed for HA. More than 80% of all HA molecules proved to be mobile, indicating that the HA clusters are not static in the timeframe of FRAP experiments (several minutes). Wild-type HA diffused somewhat slower compared to HA with deleted raft-targeting signals, but its diffusion rate was elevated to non-raft HA values after disruption of rafts by depletion of cholesterol [48]. However, HA (with or without raft-targeting signals) diffused much slower compared to the marker of inner leaflet rafts indicating that they do not diffuse together in a stable raft complex [42].

Yet, the diffusional mobility as measured by FRAP is determined by the type of transmembrane anchorage rather than raft localization: proteins anchored by lipid moieties (prenylation, S-acylation) diffuse quicker than transmembrane proteins regardless of whether they associate with rafts; the raft protein HA shows similar diffusion behavior as the non-raft protein G from vesicular stomatitis virus (VSV-G) [49]. 

FRAP is only suitable to determine the overall mobility of HA over a large area of the plasma membrane (several **μ**m^2^), which contains both raft and non-raft domains. To dissect the diffusional behavior of HA on the very small spatial and temporal scale of (undisturbed) rafts, methodology of very high resolution needs to be employed [50]. Indeed, the nanoscopic method FPALM showed that HA is mobile when observed at high spatial resolution (<200 nm), that is, at the dimension of the HA cluster. It was concluded that the membrane enwrapping the HA cluster is not composed of a solid phase and thus allows HA molecules to diffuse and eventually leave the boundary of the cluster [41]. 

No studies have been done on the clustering behavior of HA in its natural habitat, the apical membrane of polarized cells, which is particularly rich in raft lipids and possibly mostly raft-like; that is, the raft domain might be the dominant, percolating phase [51]. In other membranes, rafts are believed to be minor domains which float like a ship in a sea of non-raft lipids, a property to which the name “raft” refers. It might well be that the different properties of apical membranes might affect clustering and diffusion of HA.

In summary, these results are consistent with a model of dynamic partitioning of HA into and out of pre-existing raft domains, which permits the protein to transiently populate raft domains, as well as to undergo diffusion outside of rafts. Alternatively, HA might transiently induce the formation of rafts, which rapidly dissipate if no further stimulus is delivered. Recent real time studies on the biogenesis of individual human immunodeficiency virus (HIV) particles have shown that five to ten minutes are required for particle assembly and additional 20 minutes for budding and release [52, 53]. Assuming that a similar time frame applies for assembly and budding of influenza virus particles, it is unclear how such unstable HA clusters can support the whole assembly process. However, it is reasonable to assume that in the context of virus infection, binding of HA to M1 and the subsequent oligomerization of the latter might further stabilize the HA clusters and serve as a nucleation site for the recruitment of viral RNPs.

### 2.4. Model Membranes to Analyze Raft Localization of HA

Raft association of HA has also been analyzed *in vitro* using model membranes, which have the advantage that their chemical composition can be accurately controlled. One suitable system is giant unilamellar vesicles (GUVs), spherically closed free-standing bilayers with a size in the range of tens of micrometers, thus having a cell-like curvature. When prepared from phospholipids, sphingomyelin (SM), and cholesterol at a molar ratio of 1 : 1 : 1, the “canonical raft mixture,” separation of the lipids into two phases occurs. The phases can be visualized by fluorescence microscopy using fluorescent lipid probes, which favor either the liquid-disordered (Ld) or the liquid-ordered (Lo), raft-like phase. Membrane proteins, chemically labeled with a fluorophore, can be reconstituted into GUVs which allows direct testing of the phase preference of a protein [54]. Surprisingly, HA, either the authentic protein purified from virus particles or a peptide representing its transmembrane region, is exclusively present in the liquid-disordered, non-raft domain [55]. However, only a few proteins considered as raft components in living cells, for example, GPI-anchored proteins, associate with the raft domains in GUVs. 

Using swelling procedures, artificial membranes can also be prepared from the plasma membrane of a cell that expresses a fluorescent construct of the protein of interest. Similarly to GUVs, these giant plasma membrane vesicles (GPMVs) show long-lasting, large-scale separation into raft and non-raft phase upon cooling, but contain the lipid and protein diversity of natural membranes. Using such membranes, partitioning of HA (fused to a fluorescent protein) was more variable; that is, a minor, but significant amount was also present in the raft-like phase [55, 56]. 

The differences in raft localization of HA in cellular membranes, GPMVs, and GUVs might be explained by differences in the packaging order of their lipids. Using fluorescent lipid probes, it was shown that the raft phase is most densely packed in GUVs, a property which might prevent access especially of transmembrane proteins [55, 57]. In addition, GUVs and GPMVs lack cortical actin which probably helps in organization and maintenance of raft domains in cells. Furthermore, lipid asymmetry of the bilayer, characteristic for the plasma membrane of cells, is not preserved in GUVs and GPMVs. Finally, in some procedures to prepare HA or GPMVs, the reducing agent dithiothreitol (DTT) is used, which is known to cleave off thioester-bound fatty acids. In the mentioned studies, this might have removed the raft-targeting feature and concomitantly have led to non-raft localization of HA. An alternative procedure for GPMV formation which avoids the usage of DTT showed that many proteins predicted to be raft-localized in cells partition into the ordered phase [35]. The study also showed that a large fraction of raft-associated transmembrane proteins is palmitoylated and that this hydrophobic modification is required for raft partitioning. It will be interesting to see how HA behaves in that artificial membrane system. 

In principle, faithful reconstitution of viral proteins into model membranes might be the first step towards an *in vitro* system for virus assembly and budding that would allow to decipher all the required components, that is, individual viral proteins and lipids.

### 2.5. Raft Localization of Other Influenza Virus Membrane Proteins

Much less is known about the raft localization of the second glycoprotein of influenza virus, the **neuraminidase NA**, which is also DRM-associated and apically transported. The signals for apical transport and raft localization are both situated in the transmembrane region of NA, but overlap only partly. Raft targeting was mapped to the TMR half situated in the outer membrane leaflet (label 3 in Figure 1(b)), but the molecular cause for this has not been determined [58, 59]. Immunoelectron microscopy showed that NA localizes to the same microdomains as HA in virus-infected cells [60]. No functional fluorescent construct of NA has been described so far that would allow to study raft association in living cells similarly to the experiments conducted with HA, for example, by FRET.

The **matrix protein M1** does not contain a transmembrane domain and is anchored to cellular membranes by a variety of interactions [14]. M1 expressed alone is not associated with DRMs, but coexpression of HA and/or NA increases detergent resistance of M1 [17, 18]. It was therefore proposed that M1 is drawn to rafts of the plasma membrane by interactions with the cytoplasmic tails of HA and NA, but such an interaction has not been directly demonstrated so far. However, viruses lacking the cytoplasmic tails of HA and NA were found to have severe assembly defects, show irregular morphology, and are defective in vRNP packaging [61]. Those defects were much less pronounced when only one cytoplasmic tail was missing indicating redundant functions of both tails [62].

The second splice product of the M gene, the **ion-channel protein M2**, is not associated with DRMs [18]. However, M2 possess two possible raft targeting features, S-acylation [63] and an affinity for the raft-lipid cholesterol [64]. Several overlapping CRAC motifs, which are thought to mediate the interaction with cholesterol, and the single acylation site are both located within an amphiphilic helix in the cytoplasmic tail of M2 (label 4 in Figure 1(b)). It was therefore proposed that acylation and cholesterol binding target the amphiphilic helix to the raft domain but the relatively short transmembrane region of M2 prevents complete immersion of the protein in the more ordered, hence thicker raft domains. As a consequence, M2 was hypothesized to localize to the edge of the viral budozone, to be involved in raft coalescence and to mediate pinching off of virus particles from the plasma membrane by the induction of curvature through wedge-like insertion of the amphiphilic helix into the membrane [64]. 

Testing possible raft localization of M2 with FRET showed that the molecule (fused to a fluorescent protein) does not interact with the double-acylated marker for inner leaflet rafts [65]. However, in GPMVs prepared in the absence of DTT, M2 (partly) partitioned into the raft domain, a property which was dependent on acylation, but not on intact CRAC motifs. Thus, in principle, M2 can interact with raft domains but an enrichment at the interface between the liquid-ordered and -disordered phase was not observed in this system [66].

Surprisingly, the results from FRET experiments point to an interaction (or very close colocalization) of M2 with HA [65]. The FRET signal between M2 and HA (fused to fluorescent proteins) depends on the raft-targeting signals of HA and on an intact actin cytoskeleton, reinforcing the notion that cortical actin is involved in the organization of the viral budozone. How can it be reconciled that M2 clusters with raft-associated HA, but not with the double-acylated raft marker? The raft marker, when expressed in the absence of HA, is probably present in small, unstimulated rafts, to which M2 has no access. HA organizes the larger viral budozone, into which the raft marker can partition; M2 apparently interacts with this functionalized domain. Thus, M2 must have an intrinsic signal that targets the protein to the viral budozone; this signal might be identical or similar to the (unidentified) signal for apical targeting of the protein. In the course of virus infection, M2 shows increasing DRM and cholesterol-rich membrane association [67]. This is most likely mediated by the matrix protein M1, which bridges the viral components in the budozone. 

There are two reports describing that the **nucleoprotein NP**, the major vRNP component, localizes to apical membranes and associates with DRMs, even when expressed in the absence of other viral proteins [68, 69]. This observation implies that NP contains intrinsic signals for apical transport and raft association, although the protein is hydrophilic and is not modified by lipid moieties. However, others have not seen polarized localization of NP in transfected cells [16].

## 3. Function of Rafts for Influenza Virus Replication

It is assumed that rafts play a decisive role at several steps during virus replication and are hence vital for virus viability. These steps include intracellular transport of viral proteins (most notably HA) to the assembly site, assembly and budding of progeny virus particles at the plasma membrane, environmental stability of the virus particles, and fusion of viral and host cell endosomal membrane upon virus entry.

### 3.1. Intracellular Transport of HA

HA is transported to the apical plasma membrane *via* the secretory pathway. Deletion of the raft-targeting sequence in the outer leaflet of its transmembrane region severely retards Golgi-localized processing of HA, such as acquisition of Endo-H resistant carbohydrates and proteolytic cleavage. In contrast, trimerization of the molecule in the ER was not affected demonstrating that the transport delay is localized to the Golgi apparatus (Engel, de Vries, Herrmann, Veit, submitted). This is in line with a recent model on the organization of vesicular transport through the Golgi, which predicts that each cisterna of this organelle contains two lipid phases, a “processing domain” enriched in glycerophospholipids and an “export domain” enriched in cholesterol and sphingolipids. Processing enzymes, such as glycosyl transferases, are mostly excluded from export domains and therefore remain trapped in the Golgi, whereas transmembrane cargo proteins preferentially partition into the export domain [70]. Thus, decreasing the access to raft-like export domains should decelerate transport of transmembrane proteins through the Golgi. Since the second signal for targeting of HA to rafts of the plasma membrane, S-acylation at cytoplasmic cysteines, had no effect on transport, the putative export domain in the Golgi differs from conventional rafts of the plasma membrane.

Membrane rafts might also be involved in further steps of HA transport. It was postulated early on that cholesterol-sphingolipid clusters form vesicles in the trans-Golgi network (TGN), which serve as carriers for these lipids and entrapped proteins to the apical plasma membrane in epithelial cells [71, 72]. This model suggests that association with raft-like membranes is a prerequisite for apical transport of HA. Indeed, HA acquires detergent resistance at a late stage during its transport to the cell surface, probably in the TGN [73], and lowering cholesterol levels blocks transport of HA from the TGN to the cell surface [74]. However, several mutations in the transmembrane region of HA have been described which block association with DRMs, but not apical transport [75]. The lipid content of plasma membrane rafts could differ from that of transport vesicles. This could be determined experimentally by purification of HA-containing transport vesicles and analysis of their lipidome [76]. In short, there is evidence that raft domains are involved in forward transport of raft-associated cargo proteins such as HA through Golgi and TGN. This is accompanied by an increasing cholesterol content along the secretory route (ER<Golgi<plasma membrane, [77]).

### 3.2. Budding of Virus Particles

The two glycoproteins of the virus, hemagglutinin (HA) and neuraminidase (NA), accumulate in rafts of the plasma membrane. M1 is then supposed to weakly bind to the cytoplasmic tails of HA and NA. Oligomerization of M1 strengthens the weak interactions with HA and NA and draws M1 to the viral budozone. M2, which is abundantly expressed at the plasma membrane, but largely excluded from virus particles, assumingly accumulates at the edge of the budozone. Finally, interactions between vRNPs and M1 initiate budding and release of virus particles [69, 78–81]. During that process, it must be ensured that most newly formed virus particles contain a complete set of vRNPs. Since cryoelectron tomography has shown that each virus particle contains a specific pattern of exactly eight, individually discernible vRNPs, a highly selective mechanism of genome packaging is currently the favoured model [82]. Short nucleotide sequences identical in every RNA segment, which are situated at the 5′- and 3′-termini of each RNA, form a terminal panhandle structure that is required for packaging into RNPs, but the individual packaging signals present in each vRNP still need to be identified [83, 84]. 

Budding is a membrane-remodeling process which entails membrane bending (induction of curvature), that leads to the formation of an Ω-shaped bud, followed by constriction of the bud's neck, and, ultimately, the disconnection of the particle (scission) owing to very close apposition and fusion of the neck membranes [85]. During the whole budding process, a part of the (almost) planar plasma membrane is converted into a spherical (highly curved) vesicle. Since the plasma membrane tends to stay flat, this shape change is an energetically unfavorable process and the required energy must be provided by interaction with proteins [86]. Principally, there are two ways how proteins can induce curvature in cellular membranes. Intrinsically curved proteins or protein oligomers can provide “scaffolding” that leads to membrane bending; partially membrane-inserted proteins or protein domains can act as a “wedge” by displacing membrane lipids in only one bilayer leaflet [86–88]. The lipids themselves can intrinsically favor curvature if they are “cone-shaped” (if they exhibit a difference in the cross-section area of the hydrophilic head group and the hydrophobic region). In addition, since a small spherical virus contains roughly 10% more lipid molecules in the outer bilayer compared to the inner bilayer [86], enrichment of outer leaflet lipids (or partial depletion of inner leaflet lipids) at the budding site will aid in membrane deformation. Finally, formation of raft domains could also aid in the process of budding. In that case, the hydrophobic mismatch and the height difference between the domains leads to a “line tension” at the domain interface. To minimize the free energy of the system, curvature is induced in the bilayer of the budozone, which may initiate or support protein-based budding [89, 90]. 

In contrast to many other enveloped viruses, it is still not unambiguously defined which of the influenza virus proteins provide the energy for membrane deformation. To experimentally determine the driving force for budding, that is, the “minimal set” of required viral proteins, the proteins in question are expressed in cells and the shedding of “virus-like particles” (VLPs)—vesicles containing the expressed viral proteins and having the same density as actual virus particles—is detected biochemically. At first, M1 was found to be sufficient for VLP production [91, 92], consistent with a budding model based on scaffold formation by the matrix protein. This might, however, have been an artifact of the expression system; in chemically transfected cells, HA and NA [93–95] rather than M1 were found to be sufficient for VLP formation. Remarkably, it was found that M1 artificially tagged with lipid anchors is targeted to the plasma membrane and is then sufficient for VLP formation [96]. In the context of virus infection, M1 can fulfill this function by being transported to the plasma membrane by the other viral membrane proteins (see above). Of these, HA and NA are also capable of triggering VLP formation on their own, albeit with increased efficiency if M1 is coexpressed [93]. When HA and NA cytoplasmic tail mutants were included in the VLPs, M1 failed to be efficiently incorporated into VLPs, consistent with a model in which the glycoproteins control virus budding by sorting to lipid raft microdomains and recruiting the internal viral core components. It has to be kept in mind, however, that VLP formation is prone to artifacts as cells tend to continuously shed vesicles that might unspecifically incorporate the overexpressed viral protein [97].

The role of rafts for virus budding has also been analyzed in the context of virus infection. Removal of the raft-targeting signal in the transmembrane region of HA decreased virus production, and there was less HA incorporated in the produced particles. This HA mutant was randomly distributed over the plasma membrane, contrary to wild-type HA. Thus, clustering of HA in rafts, as described above for wild-type HA, ensures its inclusion in particles and/or is required for efficient budding [98].

Likewise, the interferon-induced cellular protein viperin increases the lateral mobility of HA by decreasing its raft association and severely inhibits the release of virus particles [99]. Many of the virions on the surface of viperin-expressing cells displayed a “daisy-chain” structure in which two or more viral particles appeared to be linked by a connecting membrane. Similar (or other) abnormal virus structures have been observed for viruses with deletions of the cytoplasmic tails of HA and NA [61]. Viruses containing HA without the two palmitoylated cytoplasmic cysteines incorporated reduced amounts of the internal components NP and M1 and also revealed defects in virus release. Surprisingly, exchange of the M1 protein by that of a different influenza virus strain restored assembly of viruses with nonpalmitoylated HA [100]. This observation links palmitoylation of HA to the matrix protein. However, similar experiments with H7-subtype HA did not reveal a defect in virus budding, but in virus entry by membrane fusion (see below, [101]). Nevertheless, the cumulative evidence just described clearly indicates that HA (and especially its S-acylated cytoplasmic tail) plays an important role in virus budding.

The ultimate step in virus budding is the scission of the virus particle from the plasma membrane. Recent evidence indicates that this is mediated by the amphiphilic helix of M2, probably acting as a “wedge.” Peptides representing the helix induced the formation of vesicles from GUVs [67]. Mutation of five hydrophobic residues in the amphiphilic helix of the M2-CT affected virus shape and virus budding [67, 102]. However, neither the CRAC motifs implied in cholesterol binding nor acylation are absolutely essential for the production of virus particles: there are virus strains in which the acylation site or intact CRAC motifs are lacking, and recombinant viruses in which the acylated cysteine [103] or parts of the CRAC motifs [104] were replaced grew similarly well as the corresponding wild-type virus, and deletion of both the CRAC motif and the acylation site simultaneously also did not affect virus production, at least in cell culture (Thaa, Wolff, Herrmann, Veit, to be published). However, attenuation of virus infectivity was observed in mice both for virus with nonacylated [105] and CRAC-disrupted [104] M2. 

In addition, it is likely that cellular proteins contribute to budding. The endosomal sorting complex required for transport (ESCRT), parts of which are involved in budding of other viruses such as HIV, seems to be dispensable for influenza virus budding [93, 106]. There is however some evidence that actin is involved especially in the formation of filamentous virus particles [38, 107]. Polymerisation of actin could provide a pushing force to extend the growing bud. Additionally, the endocytic recycling GTPase Rab11 was recently identified as a budding cofactor [108]. It was subsequently shown that Rab11 (and the underlying vesicular transport pathway) is involved in cytoplasmic transport of vRNPs to the plasma membrane [109]. It will be interesting to decipher its exact mode of action as well as to identify possible other cellular budding factors. 

**Figure 2:**
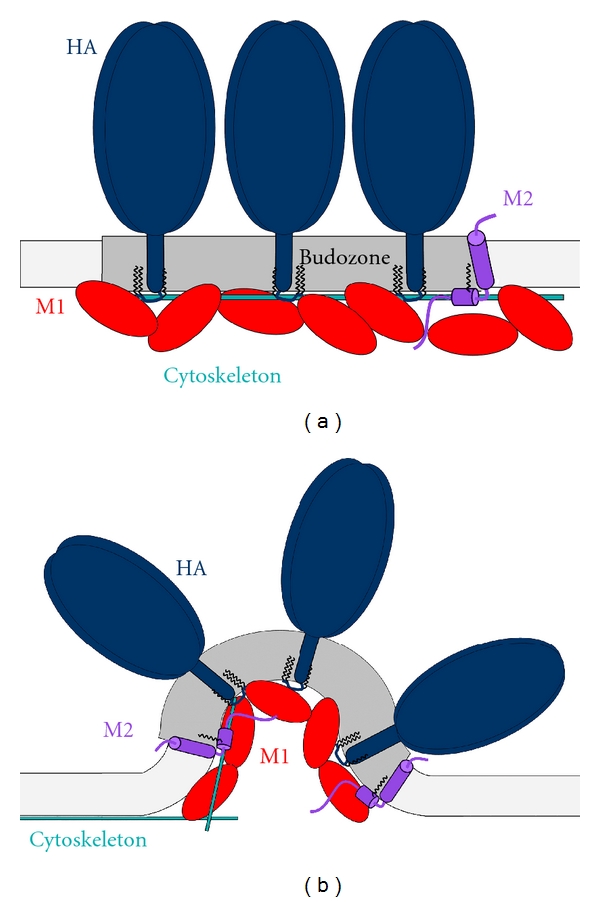
Schematic representation of influenza virus budding. (a) Formation of the budozone, a coalesced raft domain, in the plasma membrane. HA (blue) and NA (not shown) are targeted to rafts; M2 (purple) might be positioned at the edge of the budozone. M1 (red) binds to membranes and all the viral proteins including the viral RNPs (not shown) and clusters the viral components. The cytoskeleton (cyan) is involved in establishment of the budozone. (b) Formation of curvature for budding. Interactions involved: M1 acts as a scaffold from beneath, the cytoskeleton provides outward pushing, the line tension at the domain boundary leads to bending, and the amphiphilic helix of M2 acts as a wedge. See text for details.

To summarize, efficient budding of influenza virus seems to come about by the combined action of scaffold-based, wedge-mediated, and domain-induced processes (see Figure 2). M1 oligomerization could provide a scaffold, the amphiphilic helix of M2 might act as a wedge, and both work in concert with the HA-induced formation of a large-scale, intrinsically bent raft domain. However, it is surprising that mutations in several protein domains suggested to be essential for virus budding, such as the cytoplasmic tails of HA and NA and large parts of M2 including its amphiphilic helix, sometimes have no or only a moderate effect on virus replication [61, 62, 103, 104, 110–113]. Thus, budding can be considered to be a particularly robust process, relatively insensitive to disturbing effects or the failure of one of the many functionalities. The precision of the assembly process, that is, how faithfully all viral elements are included into one particle, is not known. The overwhelming majority of released particles (90–99%) are noninfectious, indicating that not all viral elements have been incorporated in a functionally active form. However, other causes for the failure to initiate infection, such as successful interference by a cellular factor, binding to an inappropriate receptor, or defects in membrane fusion, certainly contribute to the high proportion of non infectious virus particles. Furthermore, highly purified virus particles contain various cellular proteins demonstrating that they were incompletely excluded from the budding site [114]. The probably high error rate of virus budding distinguishes it from budding of cellular transport vesicles, which otherwise follow similar principles [115].

### 3.3. Stability of the Viral Envelope

It has been observed early on that the influenza virus envelope contains detergent-insoluble and ordered lipid assemblies [116]. Recently, a detailed comparison of all the lipid species present in virus particles and in the apical membrane of epithelial host cells revealed that cholesterol and sphingolipids are enriched in the viral membrane providing conclusive evidence that viruses bud through raft domains (Mathias J. Gerl and Kai Simons, personal communication). The question arises whether the raft lipids in the envelope are just a nonfunctional by-product of virus budding through rafts or whether they serve a specific function during subsequent steps of the viral life cycle.

A recent NMR study on the mobility of lipids in the viral envelope suggests that the raft lipids might be important for airborne transmission of viruses between individuals [117]. The lipids form both ordered (raft-like or solid) as well as disordered phases, but their relative proportion is strongly dependent on the temperature. At 4°C, the envelope is almost entirely in the ordered phase, which, however, is only a significant, but minor fraction at 41°C. The occurrence of two phases, probably caused by different lipid assemblies, might explain why HA and NA spikes are not randomly distributed in the viral envelope—rather, local clusters of NA spikes, surrounded by the more abundant HA, are observed by cryoelectron tomography [69, 118]. 

Thus, inclusion of raft lipids into the viral envelope equips the particle with a versatile system that autonomously regulates the rigidity of the membrane to fit the respective physiological needs. After discharge of a virus particle from the lungs of an infected person, the virus is exposed to lower temperatures. This leads to solidification of the viral envelope to protect the viral genome against environmental damage. In accordance with this, cold conditions favour transmission of influenza virus explaining its predominant winter spread [119]. After uptake in the body of the next individual, the particle is exposed to increasing temperatures which “melt" the viral envelope. A liquid membrane is required to allow the fusion of virus particles with cellular membranes.

### 3.4. Membrane Fusion Activity of HA

Membrane fusion is divided into several stages: lipid-mixing (hemifusion) and reversible formation of a fusion pore precedes the final merger of viral and cellular membranes. These events can be measured separately by loading erythrocyte ghosts with fluorescent dyes, either the membrane with lipid dyes or the interior with aqueous dyes, and following their transfer into HA-expressing cells with fluorescence microscopy [6, 120–122].

To analyze a possible effect of rafts on membrane fusion, HA without raft-targeting signals was expressed in eukaryotic cells and the fusion activity was recorded as syncytium formation (i.e., fused cells with more than one nucleus) between HA-expressing and neighboring cells. Cells expressing HA with a deleted raft-targeting signal at the beginning of the TMR were capable to induce both hemifusion and full fusion, but the number of fusion events was reduced. Virus particles containing non-raft HA were less infectious and exhibited reduced fusion activity. However, since these particles contained less HA, it was concluded that not the fusion activity *per se* was compromised but that rafts concentrate HA for efficient fusion activity [98]. 

Confusing and partly inconsistent data on the effect of removal of acylation sites on the fusion activity of HA have been published. It was reported that nonacylated H1- and H7-subtype HA and HA of influenza B virus show restricted fusion pore formation [101, 123, 124] and that nonpalmitoylated HA of the H2 subtype revealed impaired syncytium formation [125]. In contrast, HA deacylation mutants from the same H2 subtype, but also from H3, and H7 subtypes mediated cell-cell fusion [126–128]. Likewise, unperturbed transfer of aqueous dyes into HA-expressing cells was observed for avian H7-subtype HA in other studies [129] and also for human H3-subtype HA [100]. However, in all cases where an effect of acylation on the membrane fusion activity of HA was reported, a late event in this process, namely, the opening, flickering, and/or dilation of the fusion pore was affected. 

Membrane fusion is believed to proceed *via* a fusion stalk, where lipids with a certain structure connect the viral envelope with the cellular membrane such that lipid exchange occurs between the outer leaflets of both membranes [120]. It is conceivable that HA-bound fatty acids might perturb the organization of the membrane lipids at this stage of the fusion process, which would then accelerate opening and/or dilation of the fusion pore and allow membrane fusion to proceed to completion. Alternatively, HA-bound fatty acids might not work directly during fusion, but attract cholesterol to the viral envelope, which could serve a specific function during membrane fusion.

A direct role of cholesterol during membrane fusion was addressed in several studies. Extraction of cholesterol from virus particles reduced their infectivity, most likely due to an inhibition of membrane fusion [130]. In a more comprehensive analysis, HA-expressing insect cells, which naturally contain low cholesterol levels, were loaded with cholesterol and fusion with erythrocytes (labeled with fluorescent dyes) was measured. Cholesterol enhanced the rate of lipid mixing (a marker for hemifusion) and the amount and extent of aqueous dye transfer (a marker for fusion pore expansion). It was concluded that cholesterol acts both at an early stage of fusion, that is, prior to fusion pore opening, and at an late stage during fusion pore expansion [131]. In principle, the fusion-promoting effect of cholesterol might be due to three, not mutually exclusive, modes of action. First, cholesterol might bind to the transmembrane region of HA thereby directing the conformational changes required for fusion. Secondly, the negative membrane curvature spontaneously induced by the sterol might promote the local bilayer bending that takes place during membrane fusion. Finally, cholesterol might increase the mobility of HA in the membrane that is required for fusion pore expansion by increasing the fraction of lipids in the fluid state.

The confusing and partly inconsistent variety of published data on the effect of acylation site removal on the fusion activity of HA suggests that the methods used are not ideally suited for this purpose. It is questionable whether syncytium formation and fusion of HA-expressing cells with erythrocytes accurately reflect entry of influenza virus into target cells. Furthermore, these methods are only semiquantitative and kinetic measurements are barely possible. In addition, the fusion activity of HA depends on its density on the cell surface [132], which can hardly be measured accurately and cannot be controlled in conventional expression systems. It would be helpful to establish an experimental fusion system composed of closely controlled amounts of purified HA (with and without raft-targeting signals) reconstituted into lipid vesicles with the authentic composition of the viral envelope and fluorescently labeled liposomes as the target membrane to quantitatively analyze the contribution of HA-linked fatty acids to fusion pore formation and its widening.

## 4. Conclusion

In summary, HA might pass through a functional “raft cycle” during replication of influenza virus. In the Golgi, HA associates with membrane rafts, which might form vesicles to facilitate transport of entrapped proteins to the apical membrane. At the plasma membrane, HA induces the formation of the viral budozone, a membrane nanodomain where assembly of viral components and exclusion of cellular proteins occur. Upon assembly of all virus components, HA might cause bending of the membrane and M2, which is supposedly attracted to the edge of the viral budozone, might mediate pinching off of virus particles. Budding of virus particles through rafts equips the particle with an appropriate lipid mixture that protects particles from environmental damage and, in the case of cholesterol, might promote membrane fusion upon virus entry. Thus, rafts are functionally indispensable for the replication cycle of influenza virus and hence perhaps a possible target for anti-influenza drugs to be developed.
